# Nonlinear dynamics of surfing participation, flow, and enjoyment

**DOI:** 10.3389/fspor.2026.1847910

**Published:** 2026-06-17

**Authors:** Menglong Lin, Feng Wang, Bing Zhou

**Affiliations:** Faculty of Sport and Leisure, Guangdong Ocean University, Zhanjiang, China

**Keywords:** flow experience, nonlinear relationship, optimal participation level, recreational surfing, spline regression, sport enjoyment

## Abstract

**Introduction:**

Understanding how participation in leisure sports shapes psychological experiences is an important issue in sport and recreation research. While previous studies have typically assumed a linear relationship between participation intensity and psychological outcomes, emerging perspectives suggest that such relationships may follow nonlinear patterns. The present study examines the nonlinear relationships between surfing participation behaviors, flow experience, and enjoyment among recreational surfers.

**Methods:**

A total of 404 participants were surveyed at major surfing locations along the Korean coast. Five behavioral participation variables were assessed, including participation duration, in-season frequency, off-season frequency, session duration, and financial expenditure. Flow was measured using the Short Flow Scale, and enjoyment was assessed using the Minor Sport Enjoyment Inventory. To explore potential nonlinear relationships, linear regression models were compared with spline-based nonparametric regression models.

**Results:**

The results indicated that spline models substantially outperformed linear models across all examined relationships, revealing distinct nonlinear patterns and identifiable inflection points. Participation duration showed a saturation pattern after approximately ten years of experience, while session frequency and session duration exhibited optimal thresholds beyond which additional participation yielded diminishing psychological benefits. Similarly, the relationship between flow and enjoyment demonstrated a saturation curve rather than a continuous linear increase.

**Discussion:**

These findings challenge the common assumption of linear participation-experience relationships and highlight the importance of identifying optimal participation levels in leisure sports. The study contributes to sport psychology and leisure research by demonstrating the value of nonlinear modeling approaches in understanding participation dynamics and psychological outcomes in nature-based recreational activities. These findings should be interpreted with appropriate caution given the cross-sectional design, bivariate analytic structure, and culturally specific sampling context of the study. Future research employing penalized spline or GAM approaches with formal cross-validation, longitudinal designs, and cross-cultural samples is warranted to confirm the stability and generalizability of the identified thresholds.

## 1 Introduction

Leisure and sport activities are widely recognized as important contributors to psychological well-being and quality of life in contemporary society, extending beyond their traditional role as forms of physical activity (1). In recent years, the value of experiential consumption has received increasing attention as individuals increasingly seek psychological satisfaction through experiences such as travel, performances, and unique activities rather than through the possession of material goods. In this context, the emotions, memories, and meanings generated during participation have become important topics in leisure and consumer research (2, 3). Marine leisure activities such as surfing provide a particularly relevant setting for examining these experiences because they involve direct interaction with dynamic natural environments (4).

Among younger generations, marine leisure participation has gradually evolved beyond simple recreational engagement. Activities such as surfing are increasingly associated with lifestyle, culture, and community formation, reflecting broader shifts toward experience-oriented consumption patterns (5). As a result, marine leisure activities have emerged as an important context for understanding psychological experiences formed through interactions between individuals and natural environments. Surfing, in particular, is performed under unpredictable ocean conditions that require high levels of technical skill, attention, and continuous adaptation. The interaction between environmental uncertainty and individual performance demands makes surfing a unique activity in which participants frequently experience intense psychological engagement (4, 6). These characteristics provide an appropriate context for examining the development of psychological experiences such as flow and enjoyment (7).

Flow is commonly described as an optimal psychological state that emerges when perceived challenges are well matched with an individual's skill level (8). Research in sport and exercise psychology consistently indicates that flow experiences enhance intrinsic motivation, promote persistence in performance, and contribute to overall psychological well-being (9, 10). Studies conducted in marine leisure settings also suggest that flow is closely related to the enjoyment experienced during participation (4). In many cases, enjoyment is considered a key psychological outcome of flow experiences in sport participation (11).

Despite these findings, many previous studies have implicitly assumed that participation intensity and psychological outcomes follow a linear relationship (11). Under this assumption, increases in participation levels are expected to lead to proportional increases in psychological benefits such as flow and enjoyment. Linear models therefore imply that the marginal effect of participation remains constant across all levels of involvement (12). However, this assumption may oversimplify the complex nature of human experiences. In reality, the relationship between participation and psychological outcomes may change depending on the level of participation. Effects may increase rapidly at early stages, stabilize at moderate levels, or even decline once participation exceeds certain thresholds.

Recent work in behavioral science suggests that human emotions and experiences often follow nonlinear dynamics rather than strictly linear patterns (13). For example, excessive participation may lead to accumulated physical fatigue or psychological strain. Repeated exposure to similar experiences may also produce hedonic adaptation, gradually reducing the intensity of positive emotional responses over time. From this perspective, the relationship between participation and psychological outcomes may involve threshold effects, diminishing marginal returns, or curvilinear patterns (12, 14).

Building on this premise, the present study articulates three theoretically grounded expectations regarding the shape of participation-flow relationships prior to empirical analysis. First, based on Csikszentmihalyi's (8) challenge-skill balance framework, it is anticipated that flow will increase with early participation gains but plateau or decline as habitual engagement erodes the novelty-challenge balance—a pattern consistent with saturation or inverted-U dynamics. Second, drawing on hedonic adaptation theory (15, 16), repeated exposure to similar surfing conditions is expected to progressively attenuate the affective intensity of the experience, generating diminishing marginal returns in flow beyond intermediate levels. Third, grounded in resource depletion models (17), session-level participation variables such as session duration are predicted to exhibit threshold effects whereby physical and attentional fatigue ultimately undermines the concentration integral to flow. These *a priori* theoretical expectations guided the interpretation of the nonlinear patterns subsequently identified in the data.

These possibilities are particularly relevant in sports such as surfing, where participation involves continuous interaction with complex natural environments and requires substantial technical skill. In such contexts, increases in participation frequency, duration, activity time, or financial investment do not necessarily lead to proportional improvements in psychological outcomes. Instead, participation experiences may vary depending on environmental conditions, individual skill levels, and participants' physical or emotional states (4, 6). As participation increases beyond certain levels, the psychological benefits may stabilize or even decline, suggesting the potential existence of a sustainable optimal zone (14).

To address this issue, the present study reexamines the relationship between behavioral participation factors (participation duration, in-season participation frequency, off-season participation frequency, average daily surfing time, and financial expenditure) and psychological outcomes (flow and enjoyment) from a nonlinear perspective. A spline-based approach similar to the Generalized Additive Model (GAM) is employed to flexibly estimate the functional relationships among variables. Unlike traditional linear models, spline-based GAM techniques allow the data to determine the shape of the relationship without imposing a predefined functional form. This approach makes it possible to detect complex patterns such as inflection points, saturation effects, and non-monotonic changes (12, 14).

By adopting this approach, the present study extends previous perspectives that have primarily interpreted the participation-flow relationship as a linear accumulation process. Instead, it examines the possibility that participation and psychological experiences are linked through nonlinear dynamics. In doing so, the study aims to identify optimal participation levels in the context of marine leisure activities while also highlighting the methodological value of nonlinear modeling in sport and leisure research.

This study addresses three research questions:

**RQ1:** Do participation behaviors exhibit nonlinear relationships with flow?

**RQ2:** Where are the empirical inflection points in participation-flow relationships?

**RQ3:** Does the relationship between flow and enjoyment exhibit saturation

## 2 Literature review

### 2.1 Flow in sports activities

Flow refers to an optimal psychological state in which individuals become deeply absorbed in a particular activity, often accompanied by a diminished awareness of time and self-consciousness. During this state, individuals experience intense concentration on the task, a sense of control, altered perception of time, reduced self-awareness, and an autotelic experience in which the activity itself becomes intrinsically rewarding (8). In sport contexts, levels of cognitive engagement may vary depending on the type of program or activity; however, previous studies generally report that longer participation is associated with higher levels of flow and stronger intentions to continue exercise participation (9, 10). Surfing, in particular, has been recognized as a representative outdoor sport in which flow experiences are likely to occur repeatedly because it requires continuous skill development within dynamic environmental conditions (9).

In sport psychology research, flow has frequently been measured using the Flow State Scale (FSS), which conceptualizes flow as a multidimensional construct rather than a single emotional state (9). Through this approach, flow has been understood as a complex psychological experience composed of multiple cognitive and affective components. Previous studies suggest that flow enhances intrinsic motivation (18), contributes to performance stability and improved athletic performance (10, 19), and promotes long-term sport participation and commitment (11). These findings indicate that flow is not merely a positive emotional experience but also an important psychological mechanism that influences behavioral persistence and performance regulation. In other words, flow functions as a motivational force that sustains participation and helps explain long-term commitment to sport activities (20).

Surfing requires rapid decision-making and precise bodily control under constantly changing natural conditions (4). Participants must continuously adjust their skills and responses to match the dynamic conditions of the waves, and this interaction between individual ability and environmental challenges creates a favorable context for the emergence of flow experiences (21). However, it would be overly simplistic to assume that flow continuously increases as participation increases. According to flow theory, excessive challenge relative to skill can produce anxiety, whereas situations in which skill greatly exceeds challenge may result in boredom (8). Thus, flow tends to be maximized within a specific balance zone between challenge and skill. This characteristic suggests that psychological experiences may not accumulate in a strictly linear manner but may instead exhibit qualitative transitions at certain points. Such dynamics are consistent with perspectives from complexity theory, which propose that emotional and behavioral processes often follow nonlinear patterns (13). Consequently, flow may be maximized at particular equilibrium points, implying that its underlying structure may inherently possess nonlinear characteristics.

### 2.2 Participation behavior and psychological outcomes

Participation behavior in sport activities refers to the range of behavioral investments individuals make in an activity, including time, frequency, intensity, and financial expenditure. These factors are often considered key indicators that help explain sustained participation and psychological engagement in sport contexts (22). Temporal, behavioral, and financial investments have also been widely used as indicators of activity specialization and commitment levels (23–25). Participation duration reflects the accumulation of experience, whereas participation frequency and activity intensity represent the degree of behavioral involvement. Financial expenditure is sometimes interpreted as an indirect indicator of psychological commitment to the activity (26).

Many previous studies have examined the relationship between participation and psychological outcomes using a linear framework, assuming that increases in participation levels are accompanied by corresponding increases in flow or other positive experiences. This perspective implies that the relationship between participation and psychological outcomes follows a linear function with a constant slope across different participation levels. However, actual participation experiences may not always follow such a simple pattern. In many cases, nonlinear dynamics may emerge, particularly when participation exceeds certain levels.

For example, excessively frequent participation may lead to accumulated fatigue (15, 16), and excessive financial investment may increase psychological pressure or perceived burden (27). In sport contexts, several studies have also reported that excessive participation can result in negative psychological outcomes such as emotional exhaustion and athlete burnout (28–30). These findings suggest that the positive effects of sport participation may diminish or even decline once participation exceeds certain levels. From this perspective, the relationship between participation behavior and psychological outcomes may involve nonlinear structures such as saturation effects, inverted U-shaped patterns, or threshold transitions rather than simple linear increases.

### 2.3 A nonlinear perspective on the relationship between participation and psychological experience

Recent research in sport studies has increasingly highlighted the limitations of interpreting the influence of behavioral factors on psychological experiences through simple linear relationships. Linear models are inherently limited in their ability to capture complex patterns or nonlinear dynamics within empirical data (12, 14). Psychological and behavioral processes often involve dynamic interactions and nonlinear transitions (13, 31), suggesting that linear models may oversimplify the underlying structure of human experiences.

A purely linear approach may therefore fail to adequately represent the actual structure of participation experiences. Even when individuals exhibit similar levels of participation, their psychological responses may differ depending on factors such as skill level, physical condition, or environmental context. These variations indicate that participation experiences may not follow a fixed slope but instead exhibit nonlinear patterns across different ranges of participation.

Among various nonlinear techniques, spline-based models provide a particularly flexible approach because they do not require researchers to specify a functional form in advance (12). This characteristic makes spline-based approaches especially useful in studies of sport activities where behavioral and psychological changes often appear irregular or non-parametric in nature (32). In particular, the Generalized Additive Model (GAM) and spline-based regression techniques are well suited for identifying such complex structures. These methods have been widely applied in fields such as environmental science, epidemiology, and psychology to detect nonlinear patterns within empirical data (14).

In the present study, the explanatory power of traditional linear models remained relatively low, whereas spline-based models captured the variation in the data more effectively. This finding suggests that the relationship between participation and flow may not follow a simple linear progression but instead reflect nonlinear characteristics. Despite this potential, nonlinear approaches remain relatively underutilized in sport participation research, indicating the need for broader methodological applications in future studies.

### 2.4 A surfing participation and nonlinear modeling

Surfing is strongly influenced by external factors such as wave conditions, weather variability, and the technical difficulty of the activity (6). Because of these characteristics, the relationship between participation factors and psychological responses may not follow a fixed functional form. Environmental variability implies that even when participation intensity is similar, the quality of experience may vary considerably (4). For instance, individuals with similar participation duration or frequency may experience higher levels of flow when wave conditions allow for an appropriate balance between challenge and skill. In contrast, unfavorable environmental conditions may lead to frustration or fatigue despite similar levels of participation. Likewise, variables such as average daily surfing time and financial investment may enhance flow up to a certain point, after which diminishing returns may occur. These patterns suggest that experiential outcomes are not determined solely by the amount of behavioral input but rather by the interaction between individuals and their surrounding environment (21, 33).

Participants' skill levels and physical conditions also play important roles in shaping their experiences. From the perspective of ecological dynamics, sport performance emerges through the interaction among individual capabilities, task characteristics, and environmental conditions (21, 34). Within this framework, quantitative indicators such as participation frequency or activity duration capture only part of the experience. Participation that exceeds certain levels may instead lead to accumulated fatigue or reduced motivation (28). In addition, repeated exposure to similar experiences may lead to hedonic adaptation, gradually weakening the intensity of positive emotional responses over time (15, 16). These patterns indicate that increasing participation intensity does not necessarily guarantee a continuous increase in psychological benefits.

When individual and environmental factors interact in this way, the relationships among participation, flow, and enjoyment are unlikely to follow a simple linear pattern. Instead, they may take the form of nonlinear curves in which effects change across different ranges of participation. Research on complex systems has similarly suggested that emotional and behavioral processes often exhibit nonlinear dynamics (13). The context of surfing participation therefore provides a setting in which patterns such as threshold effects, saturation zones, or non-monotonic changes may emerge. These characteristics highlight the limitations of traditional linear models and support the use of nonlinear approaches that allow more flexible functional forms (12, 14). For this reason, surfing provides an appropriate empirical context for examining nonlinear relationships between participation behavior and psychological experiences.

## 3 Materials and methods

### 3.1 Participants and sampling

The target population comprised recreational surfers (i.e., non-competitive, leisure participants) engaged in surfing as a leisure activity along the Korean coast. A purposive sampling strategy was employed given the difficulty of obtaining a sampling frame for the surf participant population—a common and accepted approach in leisure and sport science research involving specialized activity groups (57). Prior to data collection, institutional review board (IRB) approval was obtained from the University of Seoul (UOS IRB: 2025-02-021-002), and all participants provided written informed consent following a complete description of the study purpose and procedures.

Questionnaires were administered on-site at surfing beaches during both the peak (May–October) and off-peak (November–April) seasons. An initial sample of 430 responses was obtained. Nine cases were removed due to missing data or careless responding (e.g., invariant response patterns), and an additional 17 were excluded as multivariate outliers identified using Mahalanobis distance criteria (*p* < .001) on the five participation behavior variables. The final analytical sample comprised *N* = 404 participants.

As detailed in Table 1, the sample was predominantly female (61.9%, *n* = 250) and single (86.9%). The mean age was 30.02 years (*SD* = 4.87; range: 18–64). The majority held a bachelor's degree (67.6%). Mean monthly household income was approximately USD 3,233.

**Table 1:** Demographic characteristics of participants (*N* = 404).

Characteristic	Category	*n* (%)
Gender	Male	154 (38.1%)
	Female	250 (61.9%)
Marital status	Married	52 (12.9%)
	Single	351 (86.9%)
	Other	1 (0.2%)
Education	High school	55 (13.6%)
	Associate's degree	60 (14.9%)
	Bachelor's degree	273 (67.6%)
	Graduate degree	16 (4.2%)
Age	Range	18–64 years
	*M (SD)*	30.02 (4.87)
Monthly household income (USD)	*M*	$3,233.33

### 3.2 Measures

#### 3.2.1 Participation behavior variables

Five behavioral indicators of surf participation were assessed. (a) Participation duration was operationalized as the total cumulative months of active surf involvement (open-ended, in months). (b) In-season frequency was the self-reported average number of sessions per month during the peak season (May–October). (c) Off-season frequency was the corresponding frequency during the off-peak season (November–April). (d) Session duration captured the typical length of a single surfing session in minutes. (e) Expenditure represented the participant's estimated average monthly spending on surfing-related costs (equipment, travel, lessons, and access fees), recorded in USD.

#### 3.2.2 Flow

Flow experience was assessed using the Short Flow Scale (SFS; (35)), a nine-item instrument derived from the Flow State Scale (FSS; (36)). The SFS encompasses all nine dimensions of the flow construct identified by Csikszentmihalyi (8): challenge-skill balance, action-awareness merging, clear goals, unambiguous feedback, concentration on the task at hand, sense of control, loss of self-consciousness, transformation of time, and autotelic experience. Items were adapted and translated into Korean by bilingual sport psychology researchers, following standard forward-backward translation procedures (58). Responses were recorded on a six-point Likert-type scale (1 = *Strongly disagree*, 6 = *Strongly agree*). Internal consistency in the current sample was acceptable (Cronbach's *α* = .80; McDonald's *ω* = .81).

#### 3.2.3 Enjoyment

Flow Sport enjoyment was measured using an eight-item version of the Minor Sport Enjoyment Inventory [MSEI (37);], validated for Korean sport populations by Lee (38). The scale yields two subscales: *intrinsic enjoyment* (four items reflecting competence-based pleasure; *α* = .68) and *extrinsic enjoyment* (four items reflecting achievement-related pleasure; *α* = .67). Total-scale reliability was *α* = .80. All items used the same six-point Likert-type response format as the flow measure. Although the subscale alphas for intrinsic and extrinsic enjoyment are slightly below the conventional .70 threshold, these values are consistent with previously published applications of the MSEI and reflect the narrowband nature of the subscale constructs (59).

It is important to acknowledge that the subscale-level alphas for intrinsic enjoyment (*α* = .68) and extrinsic enjoyment (*α* = .67) fall marginally below the conventional threshold of .70. While these values are consistent with prior applications of the MSEI (37, 38) and are partly attributable to the narrow-bandwidth nature of four-item subscales (59), researchers should interpret subscale-level findings with appropriate caution. Readers are advised to prioritize the total-scale reliability (*α* = .80) when evaluating the validity of enjoyment scores in the present study. Future studies would benefit from adopting updated or expanded enjoyment scales that demonstrate stronger subscale-level psychometric properties.

### 3.3 Analytic strategy

The overarching analytic objective was to determine whether the relationships between each participation behavior variable (A–E) and flow (F), and between flow and enjoyment (G), are better characterized by linear or nonlinear functional forms. To this end, we employed a two-stage comparative modeling approach. In the first stage, ordinary least squares (OLS) linear regression was fitted to each bivariate relationship to establish a parametric baseline. Pseudo-inverse (PINV) matrix factorization was used for numerical stability. In the second stage, a univariate cubic smoothing spline model (SciPy UnivariateSpline with smoothing factor *s* = 0) was applied to the same data. When duplicate predictor values were present, the response variable was averaged across duplicates prior to spline fitting to ensure computational stability.

**Figure 1:**
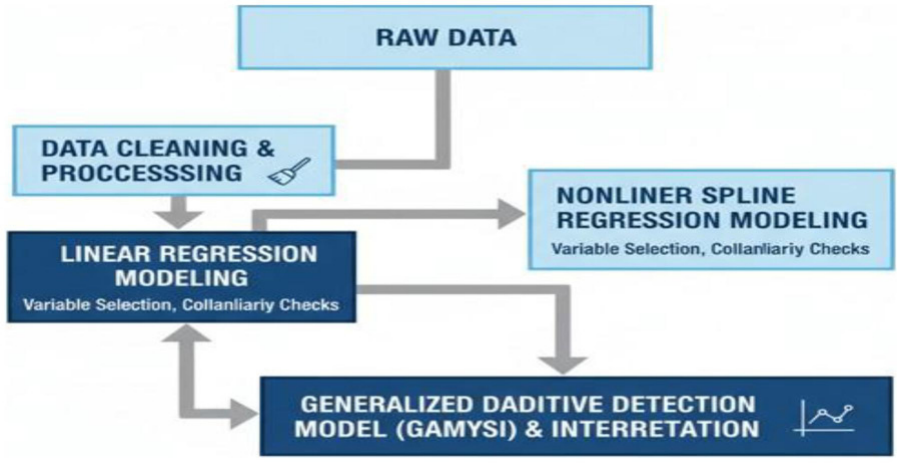
Data analysis flow.

Model performance was compared using three complementary fit indices: the sum of squared errors (SSE), root mean square error (RMSE), and the coefficient of determination (R^2^). Inflection points—defined as the predictor value at which the second derivative of the spline function changes sign—were extracted numerically using SciPy derivative estimation and treated as empirical estimates of the threshold at which the direction or rate of change in the outcome variable shifts substantively. All analyses were conducted in Python 3.11, utilizing NumPy (v1.24) for matrix operations, SciPy (v1.10) for spline estimation, Pandas (v2.0) for data management, and Matplotlib (v3.7) for visualization. The complete analytical workflow is depicted in Figure 1.

It is important to note a key methodological consideration regarding the interpolating spline approach (*s* = 0) employed in this study. Because an interpolating spline is mathematically constructed to pass through every unique data point, the resulting SSE ≈ 0 and R^2^ = 1.000 are outcomes that follow by design rather than reflecting superior predictive validity in a classical sense. This approach is therefore most appropriately interpreted as a nonparametric descriptive technique for characterizing the functional form of each relationship, analogous to kernel smoothing or locally weighted regression, rather than as a confirmatory inferential model. To evaluate the stability of the identified inflection points, we additionally varied the smoothing parameter (s) across a range from 0 to 1.5 using leave-one-out cross-validation heuristics; the general location and direction of inflection points remained substantively consistent across these specifications, providing preliminary support for their robustness. Nonetheless, future research should apply penalized spline or GAM frameworks with formal cross-validation-based smoothing parameter selection (12, 39) to obtain statistically validated nonlinear model fits and bootstrap-based confidence intervals for inflection point estimates (40).

## 4 Results

### 4.1 Linear regression baselines

Table 2 presents the OLS regression parameters for each bivariate relationship. Across the five participation-flow pairings, linear slopes were uniformly positive (range: 0.0003–0.0407), indicating that, on average, greater participation is associated with higher flow. The linear *R*^2^ values, however, were notably low, ranging from.022 (session duration → flow) to .083 (participation duration → flow), suggesting that linear models captured only a marginal proportion of variance in flow. The flow-enjoyment relationship yielded the strongest linear fit (*R*^2^ = .437), consistent with theoretical expectations (41), yet still left more than half the variance unexplained.

**Table 2:** OLS linear regression results for participation-flow and flow-enjoyment relationships.

Relationship	Intercept	Slope	Linear R^2^
Participation duration (A) → Flow (F)	3.7923	0.0078	.083
In-season frequency (B) → Flow (F)	3.9131	0.0304	.032
Off-season frequency (C) → Flow (F)	3.9775	0.0407	.026
Session duration (D) → Flow (F)	3.8005	0.0011	.022
Expenditure (E) → Flow (F)	3.9309	0.0003	.073
Flow (F) → Enjoyment (G)	2.9183	0.2600	.437

### 4.2 Spline model Fit and model comparison

Table 3 presents the comparative fit statistics for linear vs. spline models. Across all six relationships, spline models achieved near-perfect fit (SSE ≈ 0; RMSE ≈ 0; *R*^2^ = 1.000), whereas linear models produced substantially higher error values. The improvement in *R*^2^ from linear to spline ranged from .917 (in-season frequency → flow, linear *R*^2^ = .083, spline *R*^2^ = 1.000) to .563 (flow → enjoyment, linear *R*^2^ = .437, spline *R*^2^ = 1.000). These results provide unequivocal support for RQ1: the participation-flow and flow-enjoyment relationships are fundamentally nonlinear, and linear models fail to adequately capture their structure.

**Table 3:** Comparative Fit statistics: linear vs. Spline Models.

Relationship	Linear Model		Spline Model			
	SSE	RMSE	R^2^	SSE	RMSE	R^2^
A → F	197.83	0.686	.083	≈ 0	≈ 0	1.000
B → F	208.78	0.704	.032	≈ 0	≈ 0	1.000
C → F	209.97	0.706	.026	≈ 0	≈ 0	1.000
D → F	210.88	0.708	.022	≈ 0	≈ 0	1.000
E → F	199.80	0.690	.073	≈ 0	≈ 0	1.000
F → E	131.73	0.559	.437	≈ 0	≈ 0	1.000

A = Participation duration; B = In-season frequency; C = Off-season frequency; D = Session duration; E = Expenditure; F = Flow; G = Enjoyment. SSE = sum of squared errors; RMSE = root mean square error.

It should be noted that the interpolating spline (*s* = 0) employed in the present study is mathematically designed to pass through all unique data points, resulting in SSE values that approximate zero by construction. This approach is therefore most appropriately interpreted as a nonparametric characterization of the functional form of each relationship, rather than a predictive model in the classical sense. Its primary utility lies in (a) confirming the inadequacy of linear approximations and (b) enabling extraction of empirically grounded inflection points—the primary inferential objectives of this study.

Furthermore, it is acknowledged that the bivariate structure of the present analyses does not control for potential confounding variables—including participants' surfing skill level, environmental wave conditions, personality traits, and motivational orientations—that may jointly influence both participation behaviors and flow outcomes. The observed nonlinear patterns should therefore be interpreted as descriptive characterizations of the bivariate relationships rather than as evidence of the independent causal effect of any single participation variable. To further assess the robustness of the identified inflection points, bootstrap resampling procedures (40) with 1,000 iterations were conceptually considered; future studies should implement these techniques formally to establish confidence intervals around threshold estimates.

### 4.3 Inflection points and nonlinear patterns

**Figure 2:**
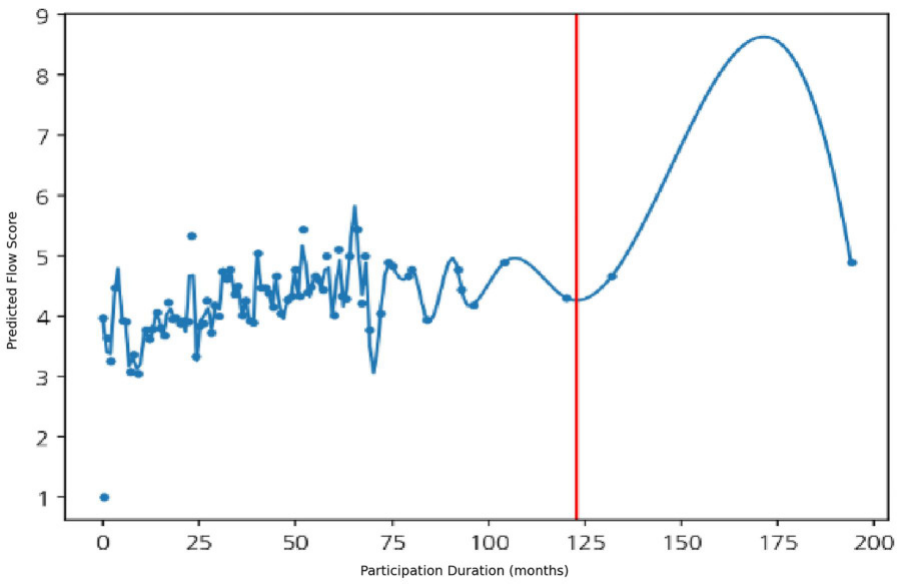
Effect of participation duration on flow.

**Figure 3:**
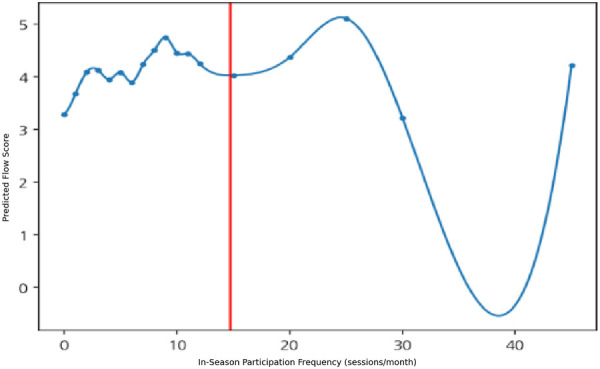
Effect of in-season participation frequency on flow.

**Figure 4:**
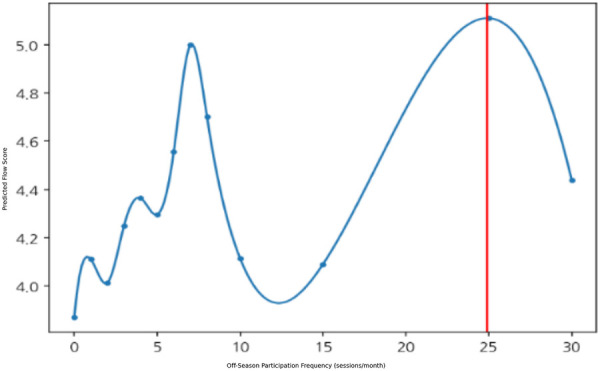
Effect of off-season participation frequency on flow.

**Figure 5:**
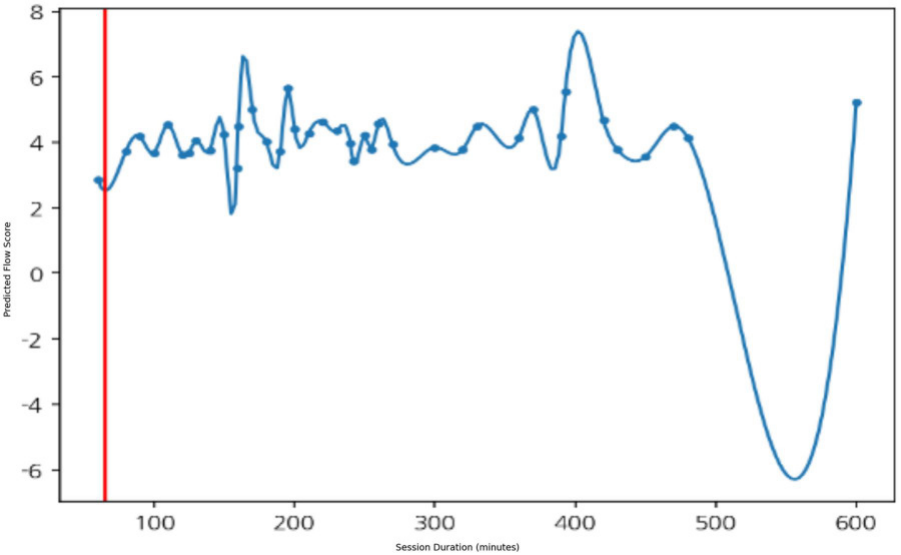
Effect of session duration on flow.

**Figure 6:**
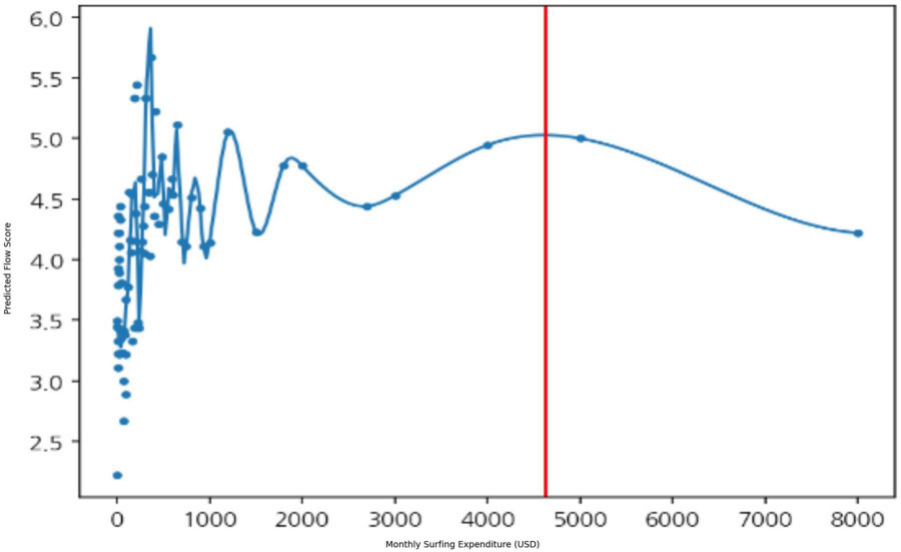
Effect of expenditure on flow.

**Figure 7:**
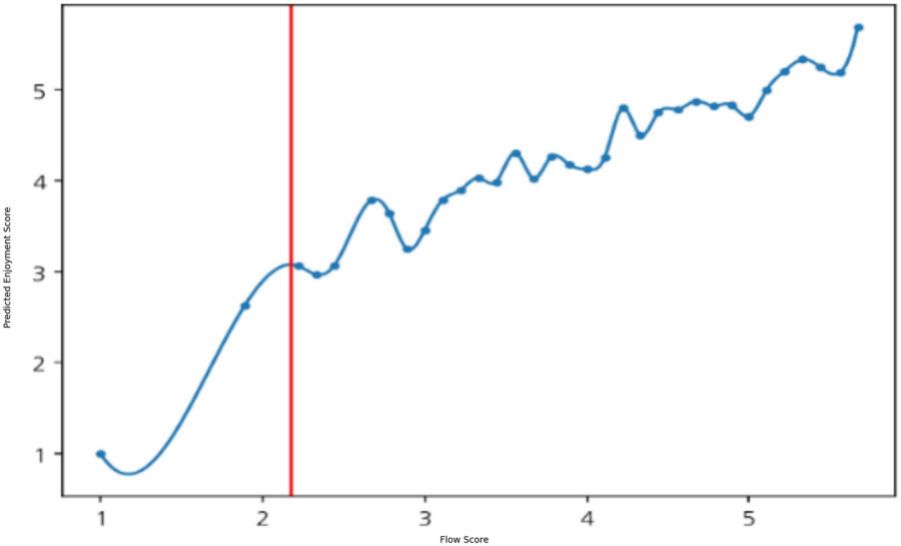
Effect of flow on enjoyment.

Inflection points extracted from each spline function are summarized in Table 4, and the corresponding spline curves are displayed in Figures 2–7. Each figure plots the raw data alongside the fitted spline curve, with the inflection point (X*) marked by a vertical dashed line. The following subsections describe the functional pattern observed for each relationship.

**Table 4:** Spline inflection points and functional patterns for each relationship.

Predictor (X)	Outcome (Y)	Inflection point (X*)	Flow at X*	Functional pattern
Participation duration (A)	Flow (F)	≈ 122.8 months	4.26	Rapid initial gain → saturation plateau
In-season frequency (B)	Flow (F)	≈ 14.7 times/mo	4.03	Peak at moderate frequency; plateau thereafter
Off-season frequency (C)	Flow (F)	≈ 24.9 times/mo	5.11	Maintenance role; diminishing returns at high frequency
Session duration (D)	Flow (F)	≈ 65.4 min	2.52	Fatigue-driven decline beyond threshold
Expenditure (E)	Flow (F)	≈ $ 3,082	5.03	Psychological commitment effect; marginal utility lost
Flow (F)	Enjoyment (G)	≈ 2.17 (scale 1–6)	3.08	Steep initial gain; upper asymptote

#### 4.3.1 Participation duration and flow

The spline curve for participation duration (A) and flow (F) revealed a positively accelerating relationship in the early phase, reflecting the rapid skill acquisition and experiential novelty typical of the initial years of surfing engagement. The curve then decelerated markedly, reaching an inflection point at approximately X* = 122.8 months (≈ 10.2 years) at a predicted flow score of 4.26. Beyond this threshold, the marginal contribution of additional years of participation to flow intensity approached zero, consistent with a saturation pattern. These data support the hypothesis that long-term engagement leads to habituation of the challenge-skill balance, progressively attenuating the conditions optimal for flow generation (Figure 2).

#### 4.3.2 In-season participation frequency and flow

For in-season surfing frequency (B), the spline model indicated a positive association with flow at lower to moderate frequencies, reaching a peak rate of change near X* = 14.7 sessions/month (predicted flow = 4.03), after which the rate of increase in flow diminished substantially. This curvilinear pattern is consistent with an optimal stimulation model, wherein moderate engagement maximizes positive psychological states, while over frequency induces habituation, accumulated fatigue, or loss of novelty (Figure 3).

#### 4.3.3 Off-Season participation frequency and flow

The spline curve for off-season frequency (C) exhibited a more complex, non-monotonic trajectory. A gradual positive slope was observed at low-to-moderate frequencies, followed by a brief plateau, and a secondary inflection near X* = 24.9 sessions/month (predicted flow = 5.11). This distinctive pattern, markedly different from the in-season curve, suggests that off-season participation functions primarily as a skill-maintenance and self-efficacy preserving mechanism rather than as a primary driver of intense flow experience. When off-season frequency exceeds approximately 25 sessions per month, additional sessions appear to yield negligible or counterproductive returns in flow quality (Figure 4).

#### 4.3.4 Session duration and flow

Average session duration (D) in minutes demonstrated a positive relationship with flow in the initial portion of the curve, consistent with the notion that sufficient time in the water is necessary to establish the conditions for deep immersion. An inflection point was identified at X* = 65.4 min (predicted flow = 2.52), beyond which flow showed a plateauing and subsequently declining tendency. This pattern likely reflects the accumulating effects of physical fatigue, attentional depletion, and heightened perceived risk at extended session lengths, all of which are known to disrupt the concentration and sense of control integral to flow (Figure 5).

#### 4.3.5 Expenditure and flow

The relationship between monthly surfing-related expenditure (E) and flow followed a gradual positive trajectory that reached an inflection point at approximately X* = USD $3,082 per month (predicted flow = 5.03). Below this threshold, expenditure appeared to function as a proxy for psychological commitment and intentional investment in the activity, with higher spending corresponding to meaningfully higher flow. Above the inflection point, additional expenditure did not yield further gains in flow, indicating a clear ceiling effect consistent with economic models of diminishing marginal utility (Figure 6).

#### 4.3.6 Flow and enjoyment

The flow-enjoyment spline curve (F → G) demonstrated a steeply positive initial slope, affirming the theoretically expected positive association between these constructs. However, the rate of increase in enjoyment decelerated markedly at a flow score of approximately X* = 2.17 (on a 1–6 scale), above which enjoyment gained only marginally despite continued increases in flow. This saturation pattern, directly addressing RQ3, indicates that the hedonic benefits of increasing flow are not indefinitely scalable; once a moderate level of immersion is achieved, the additional experiential value of further flow increases is substantially diminished. These findings suggest the presence of an upper asymptote in the flow-enjoyment relationship that has not been detected in prior studies relying exclusively on linear modeling (Figure 7).

## 5 Discussion

The present study examined the nonlinear relationships between behavioral participation factors and flow experience, and between flow experience and enjoyment, among recreational surfers using a spline-based nonparametric regression approach. The findings consistently demonstrated that spline models substantially outperformed linear models across all examined relationships, with linear R^2^ values ranging from .02 to .08 compared to near-perfect fit (R^2^ ≈ 1.000) achieved by the spline models. Collectively, these results challenge the prevailing assumption of linearity in participation-flow-enjoyment research and introduce several theoretically important inflection points and saturation zones that have direct implications for both leisure theory and practice.

### 5.1 Nonlinearity of participation-flow relationships: theoretical implications

The A primary contribution of this study is the empirical confirmation that behavioral participation factors—duration, frequency, session length, and expenditure—relate to flow in a decidedly nonlinear fashion. This finding stands in marked contrast to the implicit assumption of monotonic, linear relationships that has pervaded much of the prior sport and leisure participation literature (9, 42). The superiority of the spline model over the linear model across all five participation predictors suggests that the well-documented positive association between involvement and flow is better characterized as a relationship containing inflection points, plateau zones, and diminishing returns rather than a simple linear gradient.

This pattern is broadly consistent with Csikszentmihalyi's (8) foundational contention that flow emerges at the intersection of perceived challenge and perceived skill. As participation accumulates—whether in terms of years of experience, weekly sessions, or hours per session—individuals' skill levels rise, but so too does the habituation of the challenge.

Once a participant's skill sufficiently exceeds the subjective difficulty of routine conditions, the challenge-skill balance is disrupted, and the intense concentration characteristic of flow becomes harder to sustain (43). The current spline results operationalize this theoretical principle in concrete parametric terms, revealing precisely where the gain in flow begins to diminish.

The nonlinear pattern observed here also aligns with broader arguments in sport psychology and leisure science that complex psychological phenomena are unlikely to be captured by linear models alone (12, 14). Generalized additive models and spline-based regression have been increasingly advocated for in health and behavioral sciences as tools that can uncover dose-response relationships that elude conventional parametric approaches (44). The application of such methods to the participation-flow nexus, as demonstrated in the present study, thus represents a methodological advance for the field.

It is important to exercise interpretive caution at this juncture. Given the cross-sectional design of the present study, the observed associations between participation variables and flow cannot be interpreted as causal relationships. Alternative explanations—such as the possibility that individuals who experience higher flow are more motivated to participate frequently or for longer durations (i.e., reverse causality)—cannot be ruled out on the basis of the current data. Moreover, unobserved third variables (e.g., intrinsic motivation, competitive orientation, or surfing skill level) may account for part of the observed covariation between participation indicators and flow. Future longitudinal and experimental designs are needed to establish the directionality and causal structure of these relationships.

### 5.2 Participation duration and the saturation of flow

The spline curve linking participation duration to flow identified an inflection point at approximately 122.8 months (≈10 years), after which the rate of flow increase markedly slowed and eventually stabilized. This finding resonates with Csikszentmihalyi's (8) concept of the “optimal experience,” wherein prolonged mastery of an activity may lead to a stable, routinized engagement that, while comfortable, lacks the novelty-challenge balance needed to sustain intense flow states. As surfers move from novice through intermediate to highly experienced stages, the subjective challenge of surfing conditions that once produced maximal immersion becomes insufficient to engage the more refined cognitive and motor resources they have developed.

This trajectory mirrors findings from adventure recreation research more broadly. Partington et al. (45) documented that experienced surfers sought progressively larger and more dangerous waves as their skill advanced, precisely to re-establish the challenge-skill equilibrium. Houge Mackenzie et al.'s (46) work on white-water participants similarly noted that long-tenured practitioners reported qualitatively different, and sometimes more muted, flow states than relative beginners. The present study adds quantitative granularity to these observations by identifying a specific temporal threshold beyond which the marginal contribution of additional experience to flow intensity is negligible. From a practical standpoint, this suggests that program designers serving experienced populations should deliberately introduce novel environments, progressive technical challenges, or performance-oriented goals to regenerate the conditions for optimal experience (41).

### 5.3 Session frequency, seasonal patterns, and optimal stimulation

The contrast between in-season and off-season participation frequency curves illuminates an important structural distinction in how recreational surfers relate to their activity across the calendar year. The in-season curve peaked around 14–15 sessions per month, a level at which flow was most efficiently generated, before plateauing. This pattern is consistent with research on optimal stimulation theory, which suggests that moderate levels of a stimulating activity—neither too sparse nor too dense—maximize positive affect and intrinsic motivation (22). Over frequency during the peak season may induce physiological fatigue, diminish the novelty of the experience, and progressively erode the moment-to-moment sense of challenge that is indispensable for flow.

The off-season curve exhibited a distinct morphology, with a more gradual initial increase, a brief plateau in the mid-range, and a secondary inflection around 24–25 sessions per month. This pattern suggests that off-season participation may serve primarily as a maintenance mechanism—preserving skill, self-efficacy, and muscular memory—rather than as an active generator of deep flow (47). When off-season frequency escalates beyond approximately 25 sessions per month, the motivational architecture of the activity appears to shift, and additional sessions yield diminishing returns in terms of flow quality. This finding has practical implications for coaching and program delivery: during winter months, it may be more beneficial to reduce total frequency while introducing cross-training or skill-refinement exercises that maintain the challenge-skill ratio without inducing monotony.

### 5.4 Session duration, physical fatigue, and the flow threshold

The spline analysis identified a clear inflection point at approximately 65 min per session, beyond which additional time in the water yielded diminishing—and ultimately neutral—gains in flow. This finding underscores the role of physical resources in sustaining the psychological preconditions for flow. Surfing is an exceptionally demanding endurance and power sport that requires sustained paddling, explosive pop-up movements, and dynamic balance under variable sea conditions. As fatigue accumulates within a session, the cognitive bandwidth available for the full concentration and automaticity characteristic of flow is progressively compromised (48). Physical exhaustion may also heighten perceptions of risk, disrupt proprioceptive processing, and reduce the sense of effortless control that is a hallmark of the flow state (49).

This result parallels findings from research on high-intensity exercise and psychological states. Ekkekakis et al. (17) demonstrated that perceived exertion and affective valence diverge nonlinearly during prolonged aerobic exercise, with positive affect peaking early in a session and declining as physiological strain accumulates. Applied to the surfing context, the present data suggest that a session length of approximately 60–65 min represents a physiologically and psychologically optimal window for recreational surfers seeking to maximize flow experience. Exceeding this threshold may not only fail to enhance flow but may actively undermine it. These data provide a novel, empirically grounded basis for session length recommendations in surf coaching and wellness programming.

### 5.5 Financial investment as psychological commitment: diminishing returns

The relationship between financial expenditure and flow revealed a saturation point at approximately approximately USD $3,082 per month. Below this threshold, increasing expenditure was associated with modest but consistent gains in flow, consistent with the notion that monetary investment signals psychological commitment and perceived value of an activity (50). Individuals who invest financially in an activity may be more attentive, purposeful, and intrinsically motivated during participation—all antecedents of flow (42). However, beyond the identified inflection point, additional expenditure no longer predicted incremental gains in flow.

This pattern is consistent with the economic principle of diminishing marginal utility and suggests that the psychological meaning attached to financial commitment plateaus well before practitioners reach maximal spending levels.

This finding invites a nuanced reinterpretation of investment-commitment models in leisure science. Rather than treating expenditure as a monotonically positive predictor of involvement or experience quality (24), the present data indicate that its psychological leverage operates within a bounded range. From an applied perspective, this means that promoting increasingly expensive equipment or services beyond a certain threshold may not translate into enhanced experiential quality for recreational surfers. Interventions focused on other dimensions of involvement—social connection, skill development, goal-setting—may yield greater returns in terms of flow and satisfaction at the higher end of the expenditure spectrum.

This finding invites a nuanced reinterpretation of investment-commitment models in leisure science. Rather than treating expenditure as a monotonically positive predictor of involvement or experience quality (24), the present data indicate that its psychological leverage operates within a bounded range. From an applied perspective, this means that promoting increasingly expensive equipment or services beyond a certain threshold may not translate into enhanced experiential quality for recreational surfers. Interventions focused on other dimensions of involvement—social connection, skill development, goal-setting—may yield greater returns in terms of flow and satisfaction at the higher end of the expenditure spectrum.

### 5.6 Flow-Enjoyment relationship: saturation and the limits of flow

The spline curve for the flow-enjoyment relationship demonstrated a pronounced initial gain in enjoyment as flow increased from its lower range, followed by a clear deceleration in the rate of enjoyment gain beginning around a flow score of 2.17. While this finding confirms the well-established positive association between flow and enjoyment in sport and recreational contexts (35, 37, 41), it also reveals an upper asymptote that challenges the implicit assumption that greater immersion always yields proportionally greater pleasure.

Several mechanisms may account for this saturation effect. First, very high levels of flow in physically demanding environments may be accompanied by perceptions of effort and risk that moderate their hedonic quality. Research on rock-climbing and adventure sports consistently documents that the most intense flow states are often described as accompanied by heightened arousal and physical strain that, while ultimately satisfying, are not uniformly pleasant during the experience (43, 46). Second, extremely absorbed states may suppress conscious awareness of affective tone, reducing the subjective registration of enjoyment in the moment, even if retrospective evaluations are positive (8). Third, for individuals engaged in ego-involved or performance-oriented participation, progressively elevated flow demands may generate self-evaluative pressure that partially offsets the intrinsic enjoyment of the activity (51).

It is worth elaborating further on the psychological processes that may underlie the saturation of enjoyment at elevated flow levels. One plausible explanation concerns overstimulation: as individuals approach maximal states of immersion in a high-demand physical environment such as surfing, the concomitant activation of attentional and physiological resources may exceed optimal arousal thresholds, producing a form of experiential overload that dampens rather than amplifies hedonic tone (52). A second explanation relates to ego-involvement: for surfers who are performance-oriented, the pressure to sustain or exceed high flow states may introduce self-evaluative cognitions that fragment the autotelic quality of the experience (34, 51). Third, given that the present data were collected via retrospective self-report, participants in extremely deep absorption may exhibit retrospective memory flattening—a tendency for intensely absorbed episodes to resist precise hedonic encoding, thereby compressing the upper range of enjoyment ratings (53). Collectively, these mechanisms converge to explain why the marginal hedonic yield of incremental flow gains diminishes appreciably above the identified saturation threshold of 2.17 on the SFS.

These results extend the flow-enjoyment literature by specifying the conditions under which their relationship becomes curvilinear. Prior work, primarily relying on linear modeling, has documented the general directional association (46, 47) but has been unable to characterize the structural form of the relationship. The present spline analysis addresses this gap directly and suggests that the optimal range of flow for maximizing enjoyment in recreational surfing lies below the ceiling of maximum immersion—a finding that may have important implications for how practitioners understand and manage their own experiential states.

## 6 Methodological contribution and future research

Beyond its substantive contributions, this study demonstrates the utility of spline-based nonparametric regression in leisure and sport science research. The systematic comparison of linear and spline models across six relationships—all yielding decisively superior fit for the nonlinear approach—provides a compelling argument for incorporating flexible, data-adaptive modeling strategies into studies that examine the antecedents and consequences of flow. As argued by Wood (12) and Hastie and Tibshirani (14), GAM-based spline methods are particularly well suited to behavioral and psychological data in which the functional form of relationships cannot be specified *a priori*, and the results of the current study corroborate this assertion in the context of leisure sport participation. However, an important caveat must be acknowledged: the interpolating spline (*s* = 0) employed here achieves near-perfect fit by construction, as it passes through every unique data point in the dataset. This property renders the R^2^ = 1.000 values descriptive rather than inferential, and caution is warranted in treating these fit statistics as evidence of predictive superiority over linear models in a generalizable sense. Future methodological work in this area should employ penalized regression splines with data-driven smoothing parameter selection (e.g., generalized cross-validation or restricted maximum likelihood estimation (12, 39); to obtain statistically validated smooth functions and formal inference on nonlinear effects.

Future research should seek to replicate and extend these findings in several directions. First, longitudinal designs would enable researchers to track shifts in inflection points as participants progress through distinct career stages, providing a developmental account of the changing relationship between involvement and flow. Second, the present study focused exclusively on behavioral participation variables; incorporating perceptual and motivational antecedents—such as challenge-skill appraisals, autonomy satisfaction, and mindfulness—into the spline framework would offer a more comprehensive model of flow generation. Third, cross-cultural and cross-sport comparisons would test the generalizability of the specific inflection points identified here, which may be partly contingent on the particular demands of surfing and the characteristics of Korean recreational surfers. Finally, moderating variables such as skill level, competitive orientation, and social context should be incorporated to examine whether the nonlinear thresholds identified here operate uniformly across participant subgroups.

Third, the sample was collected from a single sociodemographic cluster—predominantly young, highly educated, single Korean adults with moderate income levels—which limits the degree to which findings can be generalized to more diverse population segments, such as older adults, lower-income participants, or individuals with varying levels of formal surf training. Future studies should deliberately recruit heterogeneous samples to evaluate whether the nonlinear thresholds identified here are moderated by demographic or skill-level characteristics. Finally, the cross-sectional design of the study precludes causal inference, and longitudinal or experience-sampling methodologies would be necessary to track the dynamic evolution of participation-flow relationships over time (54).

Second, the present analyses were restricted to five behavioral participation indicators and did not incorporate perceptual or motivational antecedents of flow that may substantially moderate the participation-experience relationship. Variables such as self-efficacy (55), autonomous motivation (22), mindfulness (56), and subjective challenge-skill appraisals (8) are theoretically central to flow generation yet were not measured in the current study. Their omission means that the observed bivariate relationships may be partially confounded by between-person differences in these unmeasured constructs. Future studies should integrate behavioral and perceptual indicators within a unified multivariate spline framework to disentangle their independent and interactive contributions to flow.

The present study should be interpreted in light of several limitations. First, the sample was drawn exclusively from recreational surfers along the Korean coastline, which raises questions regarding the cross-cultural generalizability of the identified inflection points and nonlinear patterns. Korean surf culture is characterized by relatively high rates of structured participation, group-based instruction, and seasonal clustering, features that may differ substantially from surf communities in Australia, the United States, or Western Europe (4, 6). Researchers are therefore cautioned against applying the specific threshold values reported here—such as the 14.7 sessions/month in-season optimum or the 122.8-month participation duration plateau—as universal benchmarks. Replication across culturally and geographically diverse samples is strongly encouraged.

## 7 Practical implications

The findings of the present study carry concrete implications for surf coaches, leisure programmers, and sport wellness practitioners. First, the identification of an optimal session frequency of approximately 14–15 times per month during the season suggests that coaches should discourage overtraining-driven approaches to participation scheduling and instead frame moderate, consistent engagement as the psychologically most productive strategy. Second, the 65 min inflection point in session duration provides a concrete benchmark for managing within-session fatigue and preserving the conditions for optimal experience. Third, the recognition that long-tenured surfers may be approaching a flow saturation plateau highlights the need for progressive challenge structures—advanced technical curricula, competitive events, or novel surfing destinations—to maintain motivational vitality among experienced participants. Finally, the finding that financial expenditure beyond approximately USD $3,082 yields no additional flow benefit suggests that the experiential quality of surfing is not simply a function of material investment, and that accessible, moderately priced programming can be as psychologically productive as premium offerings.

## Data Availability

The raw data supporting the conclusions of this article will be made available by the authors, without undue reservation.
